# Bystander or No Bystander for Gene Directed Enzyme Prodrug Therapy

**DOI:** 10.3390/molecules14114517

**Published:** 2009-11-10

**Authors:** Gabi U. Dachs, Michelle A. Hunt, Sophie Syddall, Dean C. Singleton, Adam V. Patterson

**Affiliations:** 1 Angiogenesis and Cancer Research Group, University of Otago, Christchurch, PO Box 4345, Christchurch 8140, New Zealand; E-Mail: michelle.hunt@otago.ac.nz (M.A.H.); 2 Auckland Cancer Society Research Centre, University of Auckland, Private Bag 92019, Auckland 1142, New Zealand; E-Mails: s.syddall@auckland.ac.nz (S.S.); deancraigsingleton@hotmail.com (D-C.S.); a.patterson@auckland.ac.nz (A-V.P.)

**Keywords:** nitroreductase, thymidine kinase, cytosine deaminase, alkylating agents, chemotherapy

## Abstract

Gene directed enzyme prodrug therapy (GDEPT) of cancer aims to improve the selectivity of chemotherapy by gene transfer, thus enabling target cells to convert non-toxic prodrugs to cytotoxic drugs. A zone of cell kill around gene-modified cells due to transfer of toxic metabolites, known as the bystander effect, leads to tumour regression. Here we discuss the implications of either striving for a strong bystander effect to overcome poor gene transfer, or avoiding the bystander effect to reduce potential systemic effects, with the aid of three successful GDEPT systems. This review concentrates on bystander effects and drug development with regard to these enzyme prodrug combinations, namely herpes simplex virus thymidine kinase (HSV-TK) with ganciclovir (GCV), cytosine deaminase (CD) from bacteria or yeast with 5-fluorocytodine (5-FC), and bacterial nitroreductase (NfsB) with 5-(azaridin-1-yl)-2,4-dinitrobenzamide (CB1954), and their respective derivatives.

## 1. Chemotherapy

### 1.1. History of Modern Chemotherapy

In 1865, as part of the first effective chemotherapy regime, chronic myelogenous leukaemia was treated with potassium arsenite [1]. However, the modern era of cancer chemotherapy only began in the early 20^th^ Century [2,3]. The 1920s saw the first major advance in chemotherapeutic treatment with researchers examining the potential therapeutic benefits of chemical warfare agents created during World War I [4,5]. One of these agents, mustard gas, produced profound lymphoid hypoplasia and myelosuppression, and during experiments aimed at producing hyperemia, its anti-neoplastic effects were discovered [6]. In 1942 a study used nitrogen mustard, an analogue of sulphur mustard gas (1,5-dichloro-3-thiapentane), to treat a patient with non-Hodgkin’s lymphoma and demonstrated for the first time that chemotherapy could induce tumour regression [7]. This sparked the search for ‘a cure’ for cancer. 

Conventional cytotoxic chemotherapy has now become standard treatment for advanced, unresectable and or metastatic disease [3]. Chemotherapy, when combined with locoregional treatment modalities such as surgery and radiotherapy, has improved the survival probability for many cancer types. For example, the median survival among patients with metastatic colorectal cancer is 5-6 months without chemotherapy; this was increased to 10-12 months with schedules containing 5-fluorouracil (5-FU) and leucovorin [8,9]. When irinotecan was subsequently included in the 5-FU and leucovorin regimen (IFL/FOLFIRI) the median survival outcome was improved to 14-17 months [10,11]. More recently, the inclusion of oxaliplatin in the 5-FU and leucovorin regimen (FOLFOX) further improved median survival to 19.5 months [12], and to 20.6 months with the addition of the anti-angiogenic agent, Bevacizumab [13].

### 1.2. Characteristics of Chemotherapy

Designing compounds that will eliminate tumour cells at concentrations which will not severely harm patients has become the main motivation for drug development in this area. The difference between tumour toxicity and host toxicity is termed the ‘therapeutic index’ and is measured as the concentration of drug required to kill a tumour cell compared to that necessary to kill a normal cell. A large index is important in creating a successful clinical agent. The majority of chemotherapies in the clinic are cytotoxic compounds, able to cause DNA damage and subsequently induce apoptosis or necrosis in tumour cells. Cytotoxic agents include alkylating compounds, antimetabolites, taxanes, platinum compounds and topoisomerase inhibitors [14,15,16,17,18]. As DNA synthesis is central to this cytotoxicity, there is evidence that rapidly dividing cells (i.e. tumour cells) are most sensitive to the effects [19]. However, even in rapidly growing tumours the proportion of cells actively cycling at any instant is low, typically 10% of the tumour cell population, leaving a significant proportion of a tumour potentially resistant to this kind of treatment [20]. 

Cytotoxic compounds do not discriminate between neoplastic cells and rapidly dividing healthy cells, such as bone-marrow (haematopoietic) precursors and gastrointestinal mucosal epithelial cells, thus leading to a range of toxic side effects such as neutropenia, thrombocytopenia, anaemia, and mucositis [21,22]. Although some supportive measures, such as G-CSF treatment and anti-nausea medications, are used in conjunction with treatment [23,24], the potential of some cytotoxic drugs to cause long-term organ damage and increased risk of leukaemias is concerning [25,26]. 

For treatment to be curative the entire tumour should be accessible as it may only require one (stem) cell to create a new tumour [27]. Most of the ~10^13^ cells in the human body are within a few cell diameters of a blood vessel. This arrangement is necessary for the delivery of oxygen and nutrients to all cells and also enables efficient delivery of medicinal agents to any (normal) cell in the body. However, solid tumours are unable to maintain this homeostatic regulation of tissue and blood vessel formation. Tumour cells can proliferate sufficiently rapidly that blood vessels are forced apart, or are unable to sprout at sufficient rates, which creates a population abnormally distant (>100 µM) from blood vessels that are subjected to chronic low oxygen conditions (hypoxia) [28]. The situation is compounded by a tumour’s poorly organised vascular architecture [29], irregular blood flow [30] and compression of blood and lymphatic vessels by tumour cells, creating additional areas of low perfusion and temporary hypoxia [31]. 

There are three key challenges to treating cells distant from blood vessels. Firstly, many chemotherapies, and in particular the cytotoxic compounds, exert selective toxicity on cycling cells, but the lack of nutrients and oxygen caused by the irregular blood vessel formation, produces a gradient of decreasing cell proliferation at increasing distance from blood vessels [32,33]. Secondly, some drugs are less active in hypoxic, acidic or nutrient-deprived microenvironments where tumour cells can thrive [29,34]. Thirdly, due to the uneven distribution of blood vessels throughout the tumour, those cells most distant are exposed to lower concentrations of drug [35]. All three challenges result in reduced efficacy of chemotherapy. To limit these problems caused by the disordered tumour vasculature, efficient biodistribution is crucial to any chemotherapeutic compound. Lipophilicity of a compound is an important determinant of its biodistribution [36].

## 2. Principals of Gene Directed Enzyme Prodrug Therapy

In an attempt to address the limitations of conventional chemotherapy, research has focused on novel approaches such as gene therapy, a form of molecular medicine, designed to introduce genetic material with selective therapeutic intent into target cells. Like cytostatic chemotherapies, the underlying assumption is that there are certain biochemical, molecular or environmental characteristics of tumour cells which distinguishes them from normal tissues, and which are able to be exploited for gene therapy. 

Gene directed enzyme prodrug therapy (GDEPT) is a gene therapy based approach which attempts to limit host toxicity by introducing new catalytic functions that pre-condition tumour cells to otherwise inert prodrugs. A prodrug is delivered to the tumour where it is converted to its active form via an exogenous enzyme, resulting in the killing of local cancer cells. This strategy can be achieved by combining the targeted and efficient delivery of a gene vector with a tissue, tumour, or condition-specific inducible promoter to restrict transgene expression to the tumour mass [37]. Preferential activation of the prodrug in gene-modified cancer cells generates toxic drug concentrations in the tumour while minimising drug exposure to normal tissues creating a wider therapeutic index [38]. 

A vast range of enzyme-prodrug combinations have been designed for use in enzyme prodrug therapy (Table 1), but few have made it through to human clinical trials. This review will concentrate on three of the GDEPT combinations which have reached clinical trials: herpes simplex virus thymidine kinase (HSV-TK) with ganciclovir (GCV), cytosine deaminase (CD) from bacteria or yeast with 5-fluorocytodine (5-FC), and bacterial nitroreductase (NfsB) with 5-(azaridin-1-yl)-2,4-dinitrobenzamide (CB1954), and their respective derivatives.

**Table 1:** Selected enzyme prodrug systems used in gene therapy (table adapted from [39,40,41,42]).

Enzyme (origin)	Prodrug	Cytotoxin	Bystander	References
**Carboxylesterase **(human, rabbit, rat)	Irinotecan	SN-38	high	[43]
	Capecitabine	5-FU		
	Paclitaxel-2-ethylcarbonate	Paclitaxel		
	dpVP-16	VP16		
**Carboxypeptidase A** (human, rat)	Methotrexate-α-peptides	Methotrexate	high	[44]
**Carboxypeptidase G2** (*Pseudomonas* R16)	CMDA	CMBA	high	[45,46,47]
	ZD-2767P	Phenol-bis-iodo nitrogen mustard		
	Self-immolative prodrugs	Alkylating agents, anthracycline antibiotics		
**Cytochrome P450 **(Human: CYP2B1, 2B6, 2C8, 2C9, 2C18, 3A),(Rat: CYP2B10), (Rabbit: CYP4B1 ±CYPOR), (Dog: CYP2B11)	Oxaza phosphorines: CPA and IFO	Alkylating agents (4-hydroxy forms)	high	[48,49]
	Acetaminophen	NABQI (*N*-acetyl benzoquinone imine)	low	
**Cytosine deaminase** (±UPRT) (*E. coli*, *S. cerevisiae*)	5-FC	5-FU	high	[50,51,52,53]
**Horseradish peroxidase** (plant: horseradish)	Indole-3-acetic acid and derivatives	3-methylene-2-oxindole	high	[54]
	Acetominophen	NABQI	low	
**NADPH-cytochrome P450 reductase** (human)	Tirapazamine, EO9, RSU1069/ misonidazole	Reduced metabolites	medium	[55]
**Nitroreductase** (*E. coli* NfsB, NfsA and other reductases)	CB1954 and analogues	Alkylating agents (N-acetoxy derivatives)	high to very high	[56]
	Self-immolative prodrugs	Alkylating agents, pyrazolidines, enediynes		
		2-fluoroadenine		
	Metronidazole	Alkylating agent	very low	[57]
**Pyrimidine nucleoside phosphorylase** (human)	5’-deoxy-5-fluorouridine	5-FU	high	[58]
**Thymidine kinase** (*Herpes simplex* virus, *Varicella zoster* virus, *Equine herpes* virus)	Modified purine and pyrimidine nucleosides: GCV, E-GCV, ACV, valacyclovir, araM, araT, BVDU	Mono phosphorylated nucleotide analogues	high, dependent on gap junctions	[59,60]
	FIAU, purine and pyrimidine nucleosides, araM	Monophosphorylated nucleotide analogues		
**Thymidine phosphorylase **(human)	Pyrimidine analogues e.g. 5’-DFUR	5-fluoro deoxyuridine monophosphate	high	[61]

Abbreviations: 5-FU (5-fluorouracil), VP16 (Etoposide), CMDA (*N,N*-(2-chloroethyl) (2-mesyloxyethyl)aminobenzoyl-L-glutamic acid), CMBA (*N,N*-(2-chloroethyl)-(2-mesyloxyethyl) aminobenzoic acid), CPA (cyclophosphamide), IFO (ifosfamide), CYP (cytochrome P450), CYPOR (cytochrome P450 reductase), NABQI (*N*-acetylbenzoquinone imine), GCV (ganciclovir), ACV (acyclovir), araM (6-methoxypurine arabinoside), araT (1-β-D-arabinofuranosylthymine), BVDU [(*E*)-5-(2-bromovinyl)-2-deoxyuridine], FIAU (1-(2’-deoxy-2-fluoro-b-D-arabino-furanosyl)-5-iodouracil), 5-DFUR (5'-deoxy-5-fluorouridine).

### 2.1. Bystander Effect

A critical problem to overcome in cancer gene therapy is the delivery of genes to a sufficient number of tumour cells to cause tumour regression [62]. This process is especially important as gene transfer efficiencies in the clinic, despite active research, are unlikely to exceed 10% of the target tissue. The bystander effect, in the context of enzyme prodrug gene therapy, is the death of non-transgenic cells, due to indirect effects of treatment of neighbouring transgenic cells, causing more wide-spread cell death than if transgenic cells alone were killed. Hence, key to current GDEPT systems is the tissue penetration capacity of the prodrug, and the subsequent ability of the activated metabolites to spread to adjacent non-transduced cells [63]. Both local and distant bystander effects have been described in aiding tumour regression. 

The level of recorded bystander effect varies between different reports and different cell lines *in vitro*. Compared to *in vivo* tumours, excess extracellular volume associated with monolayer cell cultures poses difficulties in quantifying bystander effects, due to an accumulation of metabolites in the bulk medium [64]. In addition, monolayers with cell densities between 10^5^ and 10^6^ cells/mL are still a thousand fold lower than the tissue density of tumours. A 3D multilayer or spheroid model, on the other hand, allows examination of the bystander effect in situations with tissue-like cell densities [54,64]. 3D models represent key aspects of the extravascular compartment of tumours, such as the presence of non-cycling cells arising through nutrient and oxygen depletion. Multilayers also test the ability of compounds to diffuse through layers of tumour tissue. The spatial heterogeneity of enzyme transgene expression is another component to consider when testing the bystander effect *in vitro*, as homogenous mixing of enzyme-expressing and non-expressing cells is unlikely to occur *in vivo*.

The distant, immune-mediated bystander effect involves a systemic intense anti-tumour inflammatory infiltrate which has been seen in regressing tumours treated with all three examples of GDEPT described here (reviewed by Portsmouth and others [42]). Death of transgenic tumour cells can stimulate recognition of tumour antigens leading to local inflammation and immune-mediated death of non-transgenic tumour cells, which is thought to be a major factor in the success of *in vivo* gene therapy [65]. 

Following initial gene therapy in immunocompetent animals, immunity to parental cells, i.e. the original non-modified cell line, but not to other syngeneic cell lines, is conferred [66,67]. A significant increase in both CD8+ and CD4+ lymphocytes has been reported in both the HSV-TK/GCV and CD/5-FC systems. This is important for metastatic cancers, which may not have been targeted by the original gene transfer. Rejection of parental cells suggests that the body may be more capable of mounting an effective immune response against cells which have seeded outside of the original tumour. It is of note that GCV causes immunosuppression by bone marrow toxicity, which may lead to an underestimation of the involvement of the immune system in mediating a bystander effect [65].

The distant bystander effect is accompanied by the transduction of neighbouring tumour endothelial cells [68], and the resulting blood vessel destruction may, in itself, lead to a reduction in the bystander effect, via a reduction of toxic spread of metabolites.

### 2.2. Arguments for a Strong Local Bystander Effect

**Figure 1:**
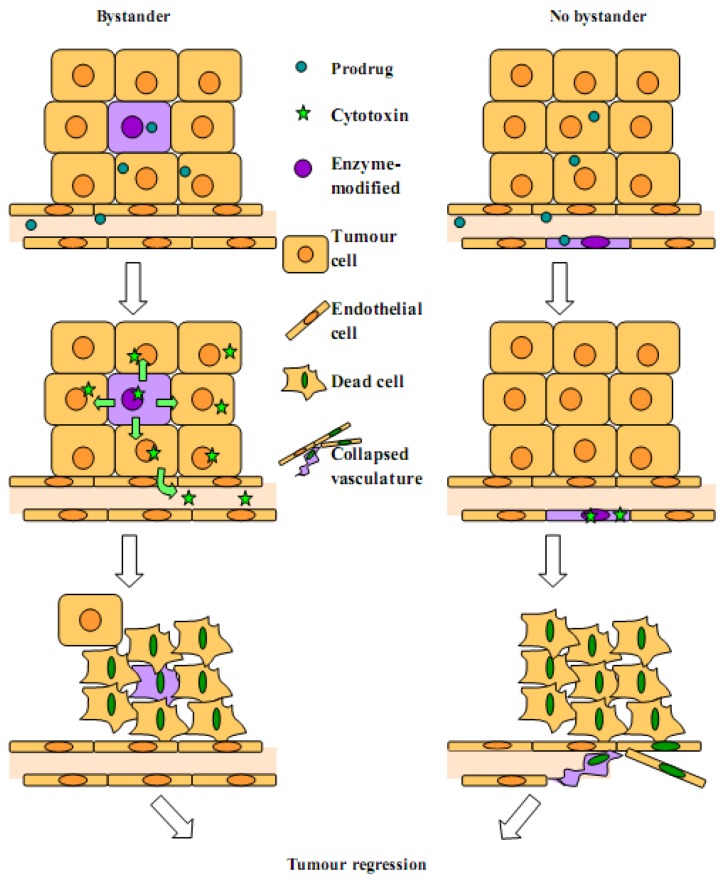
Gene directed enzyme prodrug therapy (GDEPT). Gene delivery to tumour or stromal cells is followed by gene expression and subsequent administration of a non-toxic prodrug. The therapeutic gene encodes an enzyme that converts the prodrug to a cytotoxin, leading to cell death. Surrounding cells may also be killed due to the local bystander effect, or, in the absence of a bystander effect, due to the collapse of the vasculature.

The local bystander effect involves the transfer of soluble toxic metabolites by diffusion or active transfer, via apoptotic vesicles or gap junctions (Figure 1). Both CD/5-FC (*3.2*) and NfsB/CB1954 (*3.3*) are examples where toxic metabolites spread to bystander cells via simple diffusion. The GDEPT system most reliant on gap junctions is HSV-TK/GCV (*3.1*). Moolten described how mixtures of HSV-TK+ and HSV-TK- cells sparsely seeded and then treated with GCV resulted in areas of surviving HSV-TK- cells [69]. However, if the mixtures were plated at high density very few HSV-TK- cells survived. This apparent need for cell contact was postulated to be due to the transfer of phosphorylated GCV between cells via gap junctions. Indeed, the bystander effect in cells with dysfunctional gap junctions treated with HSV-TK/GCV was severely compromised [70]. Hence, although the GCV prodrug can passively diffuse into target cells, the cytotoxic GCV-triphosphate is highly charged preventing diffusion from the cell of origin. Gap junctions allow the transfer of the GCV-monophosphate derivative to neighbouring cells where it is converted to active GCV-triphosphate by cellular kinases [71].

The reliance on gap junctional intercellular communication for the bystander effect is an imperfect strategy, since loss of this communication and reduced expression of connexins has been associated with neoplastic transformation, and can further be decreased by hypoxia [72,73,74]. For example, hypoxia/reperfusion has been shown to be a characteristic of the tumour endothelium lining blood vessels [75,76], and endothelial cell gap junctions, forming part of their barrier function, are destroyed by hypoxia/reperfusion injury [77]. To counteract this problem, co-expression of connexins Cx32 and Cx26 was utilised to increase HSV-TK/GCV mediated bystander effects in culture [70,78]. Alternatively, treatment with retinoic acid, which upregulates Cx43, led to an increased bystander effect [79].

Aside from gap junctions, soluble factors also contribute to enhanced tumour cell destruction in HSV-TK/GCV. For example, GCV metabolite-containing apoptotic vesicles from HSV-TK-transfected cells have been shown to be phagocytosed by non-transfected cells, leading to cell kill and a bystander effect [63]. 

A potential method for increasing the bystander effect is the use of the HSV-1 structural protein VP22 [80]. The VP22 protein is unusual in that cells infected with HSV-1 show localisation of the VP22 protein in a diffuse pattern in the cytoplasm. VP22 is able to spread to surrounding cells (up to 200 per infected cell), where it is taken up and selectively transported to the nucleus. Fusion proteins with VP22 are similarly transported to the nucleus of neighbouring cells [81], but not all studies utilising fusion constructs were able to demonstrate this transport property [82]. As the main cytotoxic target of most activated cytotoxins is contained within the cell nucleus, nuclear localisation of the activating enzyme could improve toxicity [81]. 

### 2.3. Arguments for a Reduced Local Bystander Effect

Cancer cells in solid tumours are often difficult to access, both for gene and drug delivery. In fact, poor transduction rates (<0.002%) and restricted geometry of vector distribution via the needle track only were the major reasons for failure in the Phase III trials (HSV-TK/GCV treatment with surgical resection and radiation in patients with glioblastoma multiforme) [62]. With these limitations in mind, enzyme prodrug combinations have been developed with the specific aim to achieve a strong bystander effect. The bystander effect is considered vital to overcome the limits and heterogeneity of gene delivery. We have proposed that this hurdle can be overcome by targeting the tumour vasculature [40]. It is a particularly attractive target for therapy, since vascular endothelial cells are easily accessible from the blood, each vessel provides oxygen and nutrients to thousands of tumour cells, and tumour vessels provide the main route for metastasis [83,84]. 

Vascular disrupting approaches, using small molecule vascular disrupting agents (VDA), have been shown to cause rapid and selective shutdown of the established tumour vasculature, leading to secondary tumour cell death [85,86]. Several small molecule VDAs are undergoing clinical testing, including the tubulin binding, microtubule depolymerizing agents, the combretastatins (lead compound CA-4-P) and 5,6-dimethylxanthenone-4-acetic acid (DMXAA) [84]. Phase I and II clinical trials confirmed selectivity of CA-4-P and DMXAA for the tumour vasculature in the clinic [84]. *In vivo*, CA-4-P caused a significant reduction in tumour blood flow within five minutes of drug administration and near complete vascular shutdown within 20 minutes [85]. Drug induced endothelial cell death was unlikely to account for these rapid changes, but morphological and functional changes associated with the endothelial cytoskeleton, leading to cell shape changes, are also likely to be involved [87].

We propose that, when targeting the tumour vasculature, the local bystander effect is not only irrelevant, but undesirable. Its absence will avoid metabolite recirculation (washout), thereby reducing systemic toxicity, whilst at the same time increasing microvascular cell kill, as the cytotoxin remains within the cell. The amplification event is achieved, but is independent of metabolite diffusion, being generated by the secondary mechanism of vascular damage-mediated tumour infarction (Figure 1). 

An example of reduced washout and increased cellular toxicity is the DNA-affinic tirapazamine (TPZ) analogue SN26955 (activated via cytochrome P450 reductase (CYPOR)), which was considerably more toxic than TPZ (400-600-fold in air and hypoxia), but failed to potentiate radiation response *in vivo*, due to limited extravascular transport [88]. Similarly, the CB1954 diol derivative, SN26634 (activated via NfsB), was extremely toxic to *nfsB*-expressing cells, but failed to induce a bystander effect in multilayers [64]. It was also a poorer substrate for the endogenous DT-diaphorase, and more soluble, due to a hydrophilic side chain, than the parent compound [89]. 

To test our hypothesis, a series of established (HSV-TK/GCV, NfsB/CB1954) and novel GDEPT combinations (NfsB/SN26634 [64], NfsB/MTZ [57], CYPOR/SN26955 and SN28141 [88], horseradish peroxidase/paracetamol [90]) were compared in endothelial cells in culture (Dachs *et al*. [91] and unpublished data). Early data demonstrated a significant cell kill in endothelial cells and an absent bystander effect for several combinations. Specifically, MTZ was highly effective in killing NfsB-modified endothelial cells, whereas adjacent control cells were spared, and superior endothelial network disruption *in vitro* was demonstrated compared with CA-4-P.

Other enzyme prodrug combinations have the potential to be used in this approach. For HSV-TK-directed GDEPT, the metabolites of pyrimidine nucleoside analogues (such as BVDU) have been shown to have inferior bystander effect compared to purine nucleoside analogues (such as GCV). Pyrimidine nucleoside analogues require HSV-TK to form both the mono- and diphosphate metabolites. The diphosphate is not transported through gap junctions as readily as the monophosphate derivative and therefore accumulates in the producer cell and does not spread as effectively [92]. Metabolites of BVDU, an agent used to treat HSV-1 infection, were less prone to transfer through gap junctions than GCV metabolites, leading to a lower bystander effect, compared to GCV [92]. 

## 3. Combinations of Enzyme Prodrug Therapy

### 3.1. Thymidine Kinase and Ganciclovir

#### 3.1.1. Mode of Action

Ganciclovir (GCV, 2-amino-9-[1,3-dihydroxypropan-2-yloxymethyl]-3H-purin-6-one) is a synthetic analogue of 2'-deoxy-guanosine first synthesized in 1980 at the Syntex Research Corporation in California as an antiviral agent [93]. GCV is phosphorylated by the thymidine kinase from HSV-1 (HSV-TK) to a monophosphate (GCV-MP), and cellular kinases complete the conversion to the active triphosphate, GCV-TP [94]. Although human cells express both cytosolic and mitochondrial TK enzymes, these endogenous enzymes have much lower ability to convert GCV compared to HSV-TK [39]. HSV-TK carries out stereospecific phosphorylation of GCV and only the (*S*)-enantiomer [and not the (*R*)-enantiomer] of the GCV monophosphate is formed [95]. GCV-TP is structurally similar to 2’-deoxyguanosine triphosphate (dGTP) and is incorporated into DNA during replication, causing inhibition of DNA polymerase, rapid chain termination and the formation of single strand breaks, leading to cell death [96]. Hence GCV is a potential human carcinogen and mutagen. GCV metabolites have a higher affinity for the HSV DNA polymerase than for the human DNA polymerase.

#### 3.1.2. Clinical Trials

HSV-TK/GCV is the only GDEPT combination to have reached phase III human trials [59,62]. Patients with glioblastoma multiforme were treated with an HSV-TK-expressing replication defective retrovirus followed by GCV and radiotherapy [62]. This treatment failed to improve patient survival over standard chemotherapy (surgery and radiotherapy). However, a subsequent phase II trial that utilised a replication defective adenovirus for delivery of HSV-TK into patients with glioma produced a clinically and statistically significant increase in median survival from 38 to 62 weeks over standard therapy [59]. Nevertheless, clinically relevant tumour regressions continue to be only sporadic and the limited biopsy data gathered during clinical trials suggest transgene expression is still a major limitation [97,98,99].

Prostate cancer has more recently been the target for HSV-TK/GCV. A phase I/II study enrolled 23 men with clinically localized prostate cancer at high risk for recurrence to receive gene therapy [100]. Data showed increased local (CD8+ cells and macrophages) and systemic immune response (CD8+ and activated CD8+, IL-12), as well as increased apoptosis and decreased microvessel density. A phase I study in eight patients with local recurrence of prostate cancer after hormonal therapy used adenoviral delivery of HSV-TK followed by GCV administration, resulting in a significant reduction of prostate-specific antigen (PSA, a marker of disease progression) [101]. 

In the setting of allogeneic hematopoietic stem cell transplantation, HSV-TK/GCV was used to control graft-versus-host disease in 23 patients treated with gene-modified donor lymphocyte infusions [102]. Immunogenicity of HSV-TK did not prevent clinical benefit.

#### 3.1.3. Enzyme Modifications and Drug Development

Because of its toxicity, efforts are underway to develop mutants of HSV-TK and derivatives of GCV. HSV-TK has a higher affinity for its natural substrate thymidine (K_m_ = 0.5 μM), than for GCV or acyclovir (ACV) (45 μM and 400 μM, respectively) [96,103,104]. Black and others described the selection of ten thymidine kinase variants from a random mutagenesis library of over a million mutant TKs, with increased activity towards GCV and ACV [105]. By further remodelling the active (nucleoside binding) site using a restricted set of random sequences, better substrate specificity towards GCV was achieved [106]. These mutants showed an increase of 5-300-fold in cellular sensitivity to GCV [106,107]. Six further mutants containing three to six amino acid changes were identified that demonstrated increased substrate specificity towards ACV, a less toxic nucleoside analogue [108]. Recently, the authors fused the mutant HSV-TK to the mouse guanylate kinase, which is the second enzyme in the GCV activation pathway, to achieve up to a 12,500-fold increase in sensitivity to GCV [109]. This improvement may allow the administration of lower, non-myelosuppressive doses of GCV to patients. Feline and canine TKs were analysed, and although they share sequence similarity with HSV-TK, their substrate specificity differed, and thymidylate kinase activity was lacking, leading to poorer cytopathic effects [110]. Secretion of HSV-TK resulted in loss of activity [111].

A number of alternative substrates for HSV-TK have been tested for GDEPT. AraT is a thymidine nucleoside, which, in the triphosphate form, has greater affinity for human DNA polymerase than the GCV or ACV metabolites [39]. However, araT did not meet the increased effective dose requirements that GCV has reached *in vitro*. The active metabolite of another pyrimidine nucleoside analogue which is converted by HSV-TK, BVDU, inhibits thymidylate synthetase in addition to causing DNA chain termination, but is more potent against varicella zoster virus (VZV) replication than HSV-1 [39]. Alternatively, circulating, liposome-encapsulated GCV causes extended release of GCV over a longer time period, and was shown to be three-fold more effective in inhibiting tumour growth compared to standard GCV administration *in vivo* [112]. E-GCV, an elaidic acid ester ‘pre-prodrug’ derivative of GCV, is much more lipophilic and stable in plasma than GCV, and is thus able to enter cells more easily by diffusion across cell membranes [113]. But it is unable to be converted by HSV-TK until the elaidic acid ester moiety is cleaved from the molecule by intracellular esterases. The resulting GCV and its phosphorylated metabolites are produced, and retained in the cell for a longer time than the phosphorylated products of GCV-treated cells. Agents able to stimulate TK activity, such as scopadulciol, have also been shown to significantly increase levels of the active, phosphorylated, metabolites of ACV and GCV, resulting in improved bystander killing [114].

### 3.2. Cytosine Deaminase and 5-FC

#### 3.2.1. Mode of Action

Fluorouracil (5-FU) is the standard chemotherapeutic agent for advanced colorectal cancer, and is used to a lesser extent to treat breast, head and neck, and pancreatic cancers [115]. Toxic side effects of 5-FU include disruption of the gastrointestinal and haematological systems, leading to myelosuppression, mucositis, dermatitis, diarrhea and cardiac toxicity. 5-fluorocytosine (5-FC) was initially synthesised at the laboratories of Roche in Basel in the early 1960s as an antimetabolite for cytosine in the search for antileukaemic drugs, but it has been used to treat fungal infections since 1968 [116]. Cytosine deaminase (CD), an enzyme found only in bacteria and fungi, catalyses the hydrolytic deamination of cytosine to uracil, and thus converts 5-FC to 5-FU [117]. Metabolism of 5-FU by intracellular enzymes leads to the production of the active metabolites 5-fluorodeoxyuridine-5’-mono-phosphate (5-FdUMP), 5-fluorodeoxyuridine-5’-triphosphate (5-FdUTP) and 5-fluorouridine-5’-triphosphate (5-FUTP). 5-FUTP is incorporated into DNA causing damage, but also prevents nuclear processing of rRNA (inhibits processing of precursors), tRNA (inhibits methylation) and mRNA (inhibits polyadenylation). 5-FdUMP irreversibly inhibits thymidylate synthase [117]. Thymidylate synthase inhibition prevents formation of 2-deoxythymidine 5’-triphosphate, so that 5-FdUTP is preferentially incorporated into DNA, leading to strand-nicked DNA and inhibition of replication, leading to cell death [118].

Unlike HSV-TK/GCV, the CD/5-FC system has a significant bystander effect that does not require direct cell contact as 5-FU can readily move out of and into cells by non-facilitated diffusion [119]. 5-FC also accumulates after multiple doses, unlike GCV. *In vivo* studies on tumours containing only 2% CD transduced cells were shown to produce significant tumour reductions [119]. *In vivo* expression of CD alone is sufficient to cause an immune response and subsequent tumour regression [120]. 

#### 3.2.2. Clinical Trials

The CD/5-FC combination has been tested in several Phase I clinical trials and patient safety was demonstrated [53,121]. Location of CD expression in patients was determined by PET imaging, using 18F-labelled 5-FC [122]. A pilot trial in three refractory cancer patients was performed, demonstrating the feasibility of delivering active *E. coli* CD via intratumoral injection of an attenuated gene-modified *Salmonella* bacterium [123]. 

#### 3.2.3. Enzyme Modifications and Drug Development

The original bacterial CD gene used for GDEPT was cloned from *E. coli* [124] and has been shown in a number of *in vitro* studies to enhance mammalian cell sensitivity to 5-FC by up to 2,000-fold [125]. CD from *Saccharomyces cerevisiae* has a higher affinity for 5-FC (K_m_ 22-fold lower) compared to the *E. coli* CD [126]. Hence, studies use either the catalytically superior yeast enzyme or attempt to improve the bacterial enzyme. Alanine-scanning mutagenesis and random mutagenesis of CD by error-prone PCR produced several CD mutants with favourable properties [127,128,129]. Recently, the bacterial CD gene was mutated via expanded random mutagenesis, resulting in improved enzyme kinetics and 19-fold shifts in substrate preference towards 5-FC, as well as improved bystander effects [130].

Uracil phosphoribosyl transferase (UPRT) is a bacterial enzyme that converts uracil to UMP, contributing to DNA and RNA synthesis, but also converts 5-FU to 5-FUMP, representing the first step in the activation pathway. UPRT has been used to sensitise cells to low concentrations of 5-FU by aiding in its conversion, therefore increasing the cytotoxic effects of 5-FC administration *in vitro* and *in vivo* [131,132]. A fusion of the CD and UPRT genes has been created resulting in greater sensitisation to 5-FC [133,134,135]. 

An 'enrichment-eradication' method to increase the bystander effect in tumours has been proposed [136]. Mice were inoculated with neuroblastoma cells carrying a fusion gene comprised of CD and UPRT, and essential *de novo* pyrimidine synthesis was blocked using *N*-(phosphonacetyl)-L-aspartate, followed by cytosine administration. Cells expressing CD/UPRT were able to survive by converting cytosine to UMP via a 'salvage pathway', but non-transgenic tumour cells had reduced survival, thereby enriching the tumour for transgenic cells. The transgenic cells were then successfully eradicated using 5-FC, leading to a 'near complete' bystander effect and decreased tumour growth *in vivo*, but no cures [136]. 

GALV, a glycoprotein from the gibbon ape leukemia virus, which causes cell fusion, was added to CD/UPRT, further increasing cytotoxicity and bystander effects [137,138]. To increase the number of cells expressing CD, and therefore to increase cell kill, fusion proteins of VP22 with CD have been constructed [139,140]. When VP22 was fused to SuperCD, a fusion of yeast CD and yeast UPRT, a significant increase in sensitivity to prodrug was reported compared to SuperCD alone [137].

### 3.3. Nitroreductase and CB1954

#### 3.3.1. Mode of Action

Another clinically investigated enzyme prodrug therapy pair is the minor nitroreductase from *E. coli*, NfsB, and 5-(azaridin-1-yl)-2,4-dinitrobenzamide (CB1954). CB1954 was developed in the 1960s at the Chester Beatty Research Institute. It was reportedly the first single agent cure of the transplanted Walker rat carcinoma 256 cell line but had little effect on other tumours known to be sensitive to difunctional alkylating agents, the presumed method of action [141]. Further research determined DT diaphorase to be the enzyme involved in metabolism of CB1954 in Walker rat carcinoma cells [142] and that it differed from the primary enzyme involved in the reduction of CB1954 in *E. coli* cells. The nitroreductase enzyme purified from *E. coli* was a flavin mononucleotide (FMN) containing flavoprotein that showed similar action to human DT diaphorase but was able to metabolise CB1954 60-fold faster [143]. Research demonstrated the enzyme to be a minor oxygen-insensitive reductase, encoded by *nfsB* [144]. 

As with 5-FU, the hydroxylamine metabolite of CB1954 is cell permeable creating a significant bystander effect without requiring direct cellular contact, although reported levels vary [57]. *In vivo* a stronger effect was reported enabling CB1954 to produce a significant growth delay in tumours containing only 5% *nfsB*-expressing cells [145]. The major species thought to be responsible for this effect is the 2-amine metabolite of CB1954 which was shown to have better diffusion properties and greater stability than the 4-hydroxylamine [146,147]. Thus, in addition to the cytotoxicity of the N-acetoxy derivative that produces DNA crosslinks, the 2-nitro reduction products are also cytotoxic as a result of the increased alkylating reactivity of the aziridine moiety.

Not all reports support the NfsB/CB1954 bystander activity. A study using NfsB/CB1954 to ablate luminal cells in the mammary gland of mice observed no *in vivo* bystander effect on closely associated myoepithelial cells [148]. The authors proposed that the NfsB/CB1954 system is therefore a better candidate for cell ablation studies than the HSV-TK/GCV combination. A follow up paper noted that NfsB-mediated cell ablation was rapid and mediated via a p53-independent apoptotic pathway [149].

In other work, a limited *in vivo* bystander effect was also reported [150]. NfsB expressed in T cells and thymocytes of mice, followed by CB1954 administration, killed both T and B cells although other non-target cells were unaffected. Both reports noted that high doses of CB1954 administration to mice caused toxicity, mostly in the form of diarrhoea, due to the presence of *nfsB*-expressing bacteria in the intestines.

#### 3.3.2. Clinical Trials

Promising pre-clinical results led to a phase I trial using CB1954, NfsB and a replication defective adenovirus CTL102 [151,152]. CB1954 toxicity limited the dose level to 24 mg/m^2^ (i.v.), giving a peak serum concentration of 6.3 ± 2.8 µmol/L and mean area under the curve of 5.8 ± 3.6 µM-h [151]. Prior work on CB1954 pharmacokinetics in mice and dogs demonstrated peak serum concentration of around 400 µM and 200 µM, respectively [153]. This data and indications from the follow up phase I/II trial established that the peak serum concentration of CB1954 in humans was not sufficient to reach active levels established in pre-clinical studies. 

More recently NfsB/CB1954 was investigated for the treatment of hip prosthesis loosening in 12 elderly patients [154]. The prostheses stabilisation was attempted by percutaneous cement injection after removal of inflammatory tissue by adenoviral delivery of NfsB andCB1954 treatment. Although there was no dose limiting toxicity, the majority of patients showed gastrointestinal and hepatic adverse effects following prodrug injection.

#### 3.3.3. Enzyme Modifications and Drug Development

A comparison of the single, double and triple mutants of NfsB produced to date identified an active mutant showing about 100-fold improved specificity with CB1954, and selectivity for the 4-nitro, rather than the 2-nitro group of CB1954 [155]. Changing the *nfsB* codon usage to mammalian preferences increased cell sensitivity to CB1954 in gene modified cells by up to 10-fold [156]. 

A recent study compared NfsA, the major *E. coli* nitroreductase, and NfsB enzymes for their ability to convert CB1954 [157]. NfsA-modified cells had up to 8-fold higher sensitivity to CB1954, relative to NfsB-modified cells. NfsB is able to reduce both the 2- and 4-nitro-positions on the prodrug, whereas NfsA preferentially reduced the 2-nitro group, leading to greater bystander effects. We have recently extended the analysis to include all eleven putative nitroreductases in *E. coli*, leading to the identification of two additional nitroreductases able to activate CB1954 (Prosser *et al*., *Biochem. Pharmacol*. in press).

To improve toxicity, secondary metabolite activation of the reactive N-hydroxylamine intermediates from CB1954, via coexpression of sulfotransferases or acetyltransferases with NfsB, was tested, and shown to increase toxicity by 16-fold [158]. However, this strategy (human NAT2 with NfsB) also significantly reduced the bystander effect. Endogenous NAT2, located in the liver and gut, may be involved in dose-limiting toxicity and side effects of CB1954 reported in patients.

The design of alternative prodrugs for nitroreductases is currently an active drug development area. Low aqueous solubility and modest kinetics of reduction by NfsB [143] have prompted development of improved analogues of CB1954 [89]. Aziridine analogues of CB1954 that were equally or more selective toward *nfsB*-expressing cells were identified. However, the increased aqueous solubility (reduced lipophilicity) of these molecules resulted in poorer bystander efficiency *in vitro* and inferior activity against *nfsB*-expressing xenografts *in vivo* [89].

Replacement of the aziridine moiety with a nitrogen mustard generated the dinitrobenzamide mustards (DNBM) prodrugs. Although originally developed as hypoxia selective cytotoxins [159], the DNBM prodrugs were subsequently found to be excellent substrates for NfsB [160]. DNBMs can be activated by NfsB and ubiquitous one-electron reductases in hypoxic cells, providing an even greater concentration of cytotoxic metabolites in the tumour. Also, since the *nfsB*-expressing and hypoxic tumour compartments are likely to be spatially distinct, activation in these subregions may provide an enhanced level of cell kill. In contrast to CB1954, the DNBM prodrugs are reduced by NfsB exclusively at the 2-nitro position to produce the 2-hydroxylamine [160]. Alterations in reactivity and therefore in key features such as cytotoxicity, solubility and *in vivo* potency can be attained by changing the substituents on the aromatic ring [161]. Analogues that can rapidly diffuse through tumour tissue to reach the hypoxic target were identified and optimised for stable, cytotoxic and lipophilic metabolites [162]. 

SN23862 (5-[*N,N*-bis(2-chloroethyl)amino]2-hydroxylamino-4-nitrobenzamide), the direct *bis*chloro-mustard analogue of CB1954, had a superior K_m_ and K_cat_, and showed increased bystander killing compared with CB1954, and was significantly more toxic when bioactivated by NfsB [160,163]. In multilayers SN23862 gave a larger differential between cell mixtures and 100% parental cell multilayers [64]. The SN23862 bystander effect was so efficient that in co-culture multilayers the non-NfsB containing cells were equally sensitive to the prodrug to those with NfsB [64]. Unlike CB1954, SN23862 and its analogues showed only weak substrate specificity for DT-diaphorase, minimising off-mechanism aerobic activation [164,165].

However, first generation DNBMs had poor aqueous solubility which limited *in vivo* application and prompted the development of DNBM phosphate esters, which act as ‘pre-prodrugs’. Systemic phosphatase activity generates the corresponding alcohols (prodrugs), which are then able to be reduced by hypoxic reductases or NfsB [166]. 

The 3,5-dinitrobenzamide-2-bromomustard, SN27686, was also shown to be more potent and selective than CB1954, produced a larger bystander effect in multilayers and showed improved *in vivo* activity in xenografts containing a minority of NfsB-modified cells [167]. Its water-soluble phosphate ester, SN28343, showed marked improvement in aqueous solubility compared to its corresponding alcohols, and demonstrated low *in vivo* toxicity, permitting a 4-7 fold molar increase in administrated dose over CB1954 [166,167]. Given its ~ 50-fold increase in dose-potency against *nfsB*-expressing cells and its markedly superior bystander effect, SN 28343 is an important advance over CB1954 [151].

Nitroaryl and nitrobenzyl phosphoramide mustard analogues of cyclophosphamide have also been described as improved prodrugs for NfsB [168,169]. Increased toxicity was reported for the lead compound, LH7 (an acyclic 4-nitrobenzyl phosphoramide), of up to 170,000-fold in NfsB-modified cells compared to unmodified cells, with similar bystander effects compared to CB1954 [169]. However, the activity of this class of prodrug against *nfsB*-expressing tumour xenografts has not been reported.

Two further classes of NfsB prodrug have been developed. The 4-nitrobenzyl carbamate prodrugs of the 5-aminobenz[e]indoline class of DNA minor groove alkylating agent, and the nitrobenzyl- and nitroimidazolyl methyl carbamate prodrugs of doxorubicin, produced examples that were selectively cytotoxic to NsfB-expressing cells *in vitro* but were found to be inferior to CB1954 *in vivo* [170,171].

An interesting prodrug for NfsB is metronidazole (MTZ). MTZ (Flagyl^TM^) is a nitroimidazole drug that has been used as an anti-protozoal and anti-bacterial agent in the clinic since the 1960s [172]. Azomycin was originally discovered by Rhone-Poulenc in Paris in a crude extract of a *Streptomyces* culture able to kill the parasite *Trichomonas vaginalis* [173], and its derivative, metronidazole, was first synthesised in 1957 (reviewed by [172]). The most interesting clinical trial was conducted in a London female prison, where inmates were isolated from the opposite sex and hence at a lower risk of re-infection of *Trichomonas*, and cure rate was 100%. Due to the careful observation by a dentist of a patient with ulcerative gingivitis, who was ‘miraculously’ cured, due to simultaneous treatment for trichomoniasis, metronidazole’s activity against anaerobic bacteria was discovered [172]. Metronidazole has remained the mainstay against anaerobic infections since.

In anaerobic bacteria, 1-electron reduction of the nitro group and the generation of short-lived reactive intermediates leads to toxicity which is reversible by oxygen [174]; however the 2-electron transfer by NfsB in air and hypoxia leads to the production of a mono alkylating agent, able to cause DNA breaks [57]. 

The NfsB/MTZ combination has been described as having no bystander effect [57], and therefore, until recently, has not been considered further for use in cancer gene therapy. The NfsB/MTZ combination has been used for cell kill of cytotoxic T lymphocytes *in vitro* [175]. More recently it has successfully been utilised to ablate specific cell lineages in zebrafish [176,177,178,179]. NfsB/MTZ may therefore be a promising combination for a gene therapy approach which does not require, or benefit from, a strong bystander effect. 

## 4. Conclusions

Gene directed enzyme prodrug therapy of cancer offers the opportunity of a targeted treatment that destroys tumours and metastases, but not normal tissues. The no-bystander approach is clearly beneficial in developmental studies to ablate single cells in particular cell lineages. However, until firm (*in vivo*) evidence has been gathered, it is not clear if the no-bystander vascular targeted GDEPT approach is likely to work in the clinic. On the other hand, the approach relying solely on bystander killing in a solid tumour mass has been developed and tested over the past 23 years, and thus far only limited clinical success has been reported. 
